# Novel lactic acid bacteria strains as inoculants on alfalfa silage fermentation

**DOI:** 10.1038/s41598-019-44520-9

**Published:** 2019-05-29

**Authors:** Mariele Cristina Nascimento Agarussi, Odilon Gomes Pereira, Rosinea Aparecida de Paula, Vanessa Paula da Silva, João Paulo Santos Roseira, Fabyano Fonseca e Silva

**Affiliations:** Department of Animal Science, Federal University of Vicosa, Viçosa, 36570-000 Brazil

**Keywords:** Plant sciences, Microbiology

## Abstract

The effects of new strains of lactic acid bacteria on alfalfa silage fermentation were evaluated. The experiment was performed using a completely randomized design (with three replicates) based on a 6 × 6 factorial assay with 6 inoculants (I): Control (CTRL), Commercial inoculant (CI), *Lactobacillus pentosus* 14.7SE (LPE), *Lactobacillus plantarum* 3.7E (LP), *Pediococcus pentosaceus* 14.15SE (PP), and *Lactobacillus plantarum* 3.7E + *Pediococcus pentosaceus* 14.15SE (LP + PP), and six fermentation periods (P): 1, 3, 7, 14, 28 and 56 days. Alfalfa was wilted for 6 h in the field, which increased the dry matter content to 368 g/kg as fed. The CP and yeast population decreased during the fermentation process. Silage inoculated with the PP strain had the lowest pH values beginning at 14 d of fermentation and the lowest acetic acid concentration on the last day of fermentation. New strains more efficiently regulated enterobacteria and mold populations at days 56 and 28, respectively. Silages inoculated with the PP strain had a higher coefficient of *in vitro* dry matter digestibility than LP silages. All of the tested novel strains resulted in positive effects on at least one chemical property of the silage during the fermentation process. However, the adding of *P*. *pentosaceus* can be indicated as the better for silage quality considering the tested treatments in the present study.

## Introduction

Forage preservation via ensiling has become a global practice because it provides a consistent, reliable, and predictable feed supply for ruminant production systems. Unavoidable losses of highly digestible nutrients caused by plant respiration, plant microbial proteolytic activity, clostridial fermentation, microbial deamination, and decarboxylation of amino acids may negatively affect conservation efficiency, increase energy and nutrient losses, and cause an accumulation of anti-nutritional compounds in silage^1^.

Alfalfa is a forage crop of great importance due to its worldwide use, high nutritional value and digestibility^2^. However, high concentrations of organic acids, salts, proteins, and minerals result in a high buffering capacity^3^. The high buffering capacity and CP, in combination with low water-soluble carbohydrates (WSC) concentrations, indicate that the ensiling properties of alfalfa are not ideal, as suggested by Muck^4^.

Therefore, the use of microbial inoculants as starters for alfalfa silage is recommended^5^. Zielińska *et al*.^6^ demonstrated that microbial inoculants altered many parameters of silages, but the strength of the effects on fermentation depended on the strain characteristics. One of the main challenges in the industry is the extent of variability in the effects of inoculant bacteria on the fermentation and preservation of silage, silage quality and animal performance, which were noted in several studies^5,7^. The lack of inoculant effects on the process may be related to the ability of the inoculated bacterium to grow rapidly in the forage mass and effectively compete with the epiphytic flora as well as the presence of adequate substrate, and it may also be related to specificities between the forage, the microorganisms present in the inoculant and the weather conditions^8^.

Muck^9^ reported that the major international companies producing inoculants are based in Europe and North America. Therefore, these products have been developed for cool-season grasses, whole-crop corn and alfalfa. The inoculants may or may not be effective when used on warm season grasses or tropical legumes, which suggests that environmental conditions affect the physiology and metabolism of the inoculated strains and may influence their effects on the fermentation process.

Oliveira *et al*.^1^ reported that *Lactobacillus plantarum* is the most commonly used silage inoculant. However, some lactic acid bacteria (LAB) species are also selected as silage inoculants because of their faster growth at high pH values (>5) compared to *L*. *plantarum*. The authors suggested that more research was needed on the effects of infrequently used LAB as individual silage inoculants on silage fermentation because little is known about their related effects on silage quality.

Inoculants containing synergistic mixtures of LAB are used via the addition of microorganisms that act during different phases of fermentation. Some *Pediococcus* strains are more tolerant to high dry matter (DM) conditions than *Lactobacillus* spp. and exhibit a wider range of optimal temperatures and pH values for growth^10^. Silages treated with one or more bacteria often have a lower pH value and acetic acid, butyric acid, and ammonia nitrogen (NH_3_-N) contents and also a higher lactic acid content and better DM recovery compared to untreated silages^5^.

Based on that, in the search for new promising strains for silage inoculants, the purpose of the present study was to investigate the effects of *L*. *pentosus* 14.7SE, *L*. *plantarum* 3.7E, *P*. *pentosaceus* 14.15SE and a mixture of *L*. *plantarum* 3.7E and *P*. *pentosaceus* 14.15SE on the chemical composition, fermentative profiles and *in vitro* DM digestibility of alfalfa silage under tropical conditions after 1, 3, 7, 14, 28 and 56 days of fermentation.

## Methods

### Location and climatic conditions

The experiment was performed between June and August 2016 at the Department of Animal Science of the Federal University of Vicosa (Viçosa, MG, Brazil), located at 20°45′S latitude, 42°52′W longitude 648 m above sea level. The annual precipitation and average temperature the year of the experiment were 1235.4 mm and 20.7 °C, respectively.

### Experimental design

The experiment was performed using a completely randomized design (with three replicates) based on a 6 × 6 factorial assay (6 inoculants × 6 fermentation periods). The periods (P) were 1, 3, 7, 14, 28, and 56 days after fermentation. The following inoculants (I) were evaluated: 1- Control (CTRL); 2- Commercial inoculant (CI); 3- *Lactobacillus pentosus* 14.7SE (LPE); 4- *Lactobacillus plantarum* 3.7E (LP); 5- *Pediococcus pentosaceus* 14.15SE (PP); and 6- *L*. *plantarum* 3.7E + *P*. *pentosaceus* 14.15SE (LP + PP). The commercial inoculant Silobac (CHR Hansen’s^®^, Hørsholm, Denmark), which contains *L*. *plantarum*, *P*. *pentosaceus*, maltodextrin, sodium aluminosilicate and whey, was used to compare its effectiveness with the new strains.

### Characterization of the inoculants

The three wild strains of LAB used in this study belong to the microorganism bank of the Forage Laboratory of the UFV and were isolated from wilted and non-wilted alfalfa silages. The sequences of the strains are deposited in the GenBank database with the following access numbers: *L*. *pentosus* 14.7SE - MH924298; *L*. *plantarum* 3.7E - MH924275; and *P*. *pentosaceus* 14.15SE - MH924301.

Growth tests at different temperatures (15 and 45 °C), pH (3.5, 4.0, 4.5 and 8.5 at 37 °C), salt concentrations (40 and 60 g/L of NaCl at 37 °C), gas production and antimicrobial activities were performed in a previously study^11^.

The efficiency in reducing pH was measured using a potentiometer after 24 h at 37 °C in alfalfa broth. Alfalfa broth was obtained from 100 g of herbage crushed in 400 ml of distilled water in an industrial blender for 1 min and was filtered and sterilized (121 °C, 15 min). The strains were activated twice in MRS broth for 24 h, and one more time in tubes containing 3 ml of the alfalfa broth for 24 h. A sample (10%) of the inoculum was added to a tube containing 5 ml of the alfalfa broth (pH = 5.87). Samples after 24 h incubation were analyzed for metabolite production (lactic, acetic and propionic acids) using HPLC (Shimadzu Scientific Instruments, Columbia, MD) according to Siegfried *et al*.^12^. The characteristics of the strains are presented in Table S1.

The strains were selected based of their metabolite production, ability to induce a fast drop in pH, growing capacity in different conditions and broad-spectrum antimicrobial activity against pathogenic and harmful spoilage organisms, such as *Listeria monocytogenes* 19117, *Listeria monocytogenes* 7644, and *Escherichia Coli* K12.

### Silage production

The harvesting of alfalfa cv. Crioula (*Medicago sativa* cv. Crioula) was performed using a costal brush when the plants were at the early bud stage. Fresh alfalfa was wilted to a DM content of approximately 368 g/kg as fed and chopped into approximately 1.5-cm length particles.

Novel strains were cultured in MRS broth for 14 h, which was the average time that showed the maximum number of cells. Each inoculum was standardized using a spectrophotometer (630 nm) at an optical density of 0.05, in 20 ml of MRS broth, and the amount needed to reach the theoretical application rate of 10^5^ colony forming units (cfu)/g of fresh weight were centrifuged at 1,000 g for 10 min. The supernatant was discarded.

Three replicated piles (each pile treated individually) containing approximately 10 kg of fresh alfalfa were prepared for each treatment (total of 18 piles). Inoculants were diluted in 15 ml of sterilized alfalfa broth plus 35 ml of water, which was sprayed uniformly on chopped forage. A total of 500 g of fresh alfalfa was packed into nylon-polyethylene bags (25 × 35 cm; Doug Care Equipment Inc., Springville, CA), and the air was evacuated from the bags using a vacuum sealer (Eco vacuum 1040, Orved, Italy). The same amount of alfalfa broth and water were applied to the CTRL silages. A total of 108 bags were prepared and stored in the laboratory at room temperature (range, 23–27 °C). Three bags from each treatment were opened 1, 3, 7, 14, 28, and 56 d after fermentation.

### Fermentative profile

Twenty-five grams of the forage and silage samples from each mini-silo were homogenized in 225 ml of sterile Ringers solution (Oxoid, Basingstoke, UK) in an industrial blender for 1 min. The aqueous extract was divided in two portions: one portion was used to measure the pH using a potentiometer and determine the concentrations of NH_3_-N^13^, WSC^14^ and organic acids, as described previously.

### Quantification of microbial populations

The second portion of the aqueous extracts was used to quantify the LAB, enterobacteria, yeast and mold populations. Serial dilutions were made in Ringers solution and plated using the plate technique in different culture media. Cultivation of the LAB population was performed on MRS agar (Difco^TM^ *Lactobacilli* MRS Agar^®^) at 37 °C for 48 h. Culture of enterobacteria was performed on VRB agar (Violet Red Bile) at 37 °C for 24 h, and the cultivation of mold and yeast was performed in Dextrose Potato Agar media containing a 1.5% tartaric acid solution (10% w/v) at 25 °C for 96 h. The cfu was determined on plates containing 25 to 250 colonies.

### Chemical composition

Alfalfa samples before ensiling and their silages were dried in a forced-air oven at 55 °C for 72 h and milled in a Willey mill with a 1-mm sieve for determination of the DM (method 934.01) and CP (method 984.13), as described by the AOAC^15^.

Acid detergent fiber (ADF)^15^, (method 973.18) and neutral detergent fiber (NDF) using heat-stable α-amylase without sodium sulfite were only analyzed in the forage (day 0) and silage samples after 56 days of fermentation and corrected for residual ash^16^. Corrections of the NDF and ADF for nitrogen compounds were performed according to Licitra *et al*.^17^.

The microbial populations and chemical composition characteristics of alfalfa forage prior the ensiling processes are shown in Table 1.

**Table 1:** Chemical composition (g/kg of DM, unless otherwise stated) and microbial populations (log cfu/g of fresh weight) of alfalfa forage before ensiling.

Item	DM (g/kg)	CP	NDF	ADF	pH	WSC	LAB	Yeast	Mold	Ent
	368.1	175.4	403.1	270.7	6.54	23.9	6.54	5.19	5.16	6.37

Item: DM = Dry matter; CP = Crude protein; NDF = Neutral detergent fiber; ADF = Acid detergent fiber; WSC = Water-soluble carbohydrates; LAB = Lactic acid bacteria; ENT = Enterobacteria.

### *In vitro* dry matter digestibility

*In vitro* DM digestibility (IVDMD) was performed on alfalfa silage samples from 56 d of fermentation. Dried 1-mm screen samples (0.5 g) were weighed in duplicates on F57 bags (Ankom Technology Corp.). Fermentation was performed *in vitro* using the DaisyII rotating jar in an incubator (Ankom Technology Corp.), according to methods described by Tilley and Terry^18^ and adapted by Holden^19^. The analyses were replicated on two different occasions.

A composited inoculum was prepared with rumen fluid and rumen solids (pH = 6.09) collected from 3 cannulated lactating Holstein cows in mid-lactation that were fed a diet containing 70 g/kg of corn silage and 30 g/kg of concentrate mix (DM basis). Bags were removed from the jars after 48 h of fermentation, rinsed, and dried in a forced-air oven at 55 °C for 48 h. The coefficients of *IVDMD* was determined.

### Statistical analysis

The data were analyzed using the MIXED procedure of SAS^®^ (v. 9.4 SAS Institute Inc., Cary, NC). The general model was given by Y_ijk_ = μ + I_i_ + P_j_ + (IP)_ij_ + e_ijk_, where Y_ijk_ = response variable; μ = overall mean; I_i_ = effect of inoculant i; P_j_ = effect of period j; (IP)_ij_ = effect of the interaction between the level i of factor I and level j of factor P; and eijk = random residual term. The estimated means were compared using Tukey’s test considering a significance level of 0.05.

## Results

**Figure 1:**
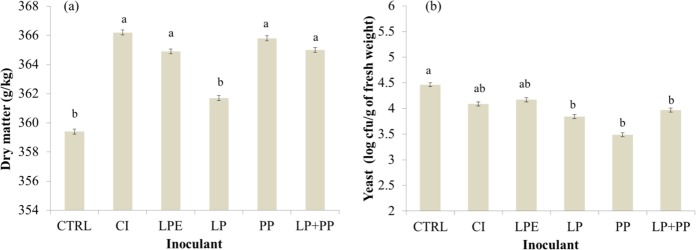
Effect of the microbial inoculants on the dry matter content (**a**) and yeast population (**b**) of alfalfa silages. ^a,b^Means followed by different letters are significantly different according to Tukey’s test (*P* < 0.05). CTRL = Control (without inoculant); CI = Commercial inoculant - Silobac; LPE = *Lactobacillus pentosus*; LP = *Lactobacillus plantarum*; PP = *Pediococcus pentosaceus*; LP + PP = *Lactobacillus plantarum* + *Pediococcus pentosaceus*.

**Figure 2:**
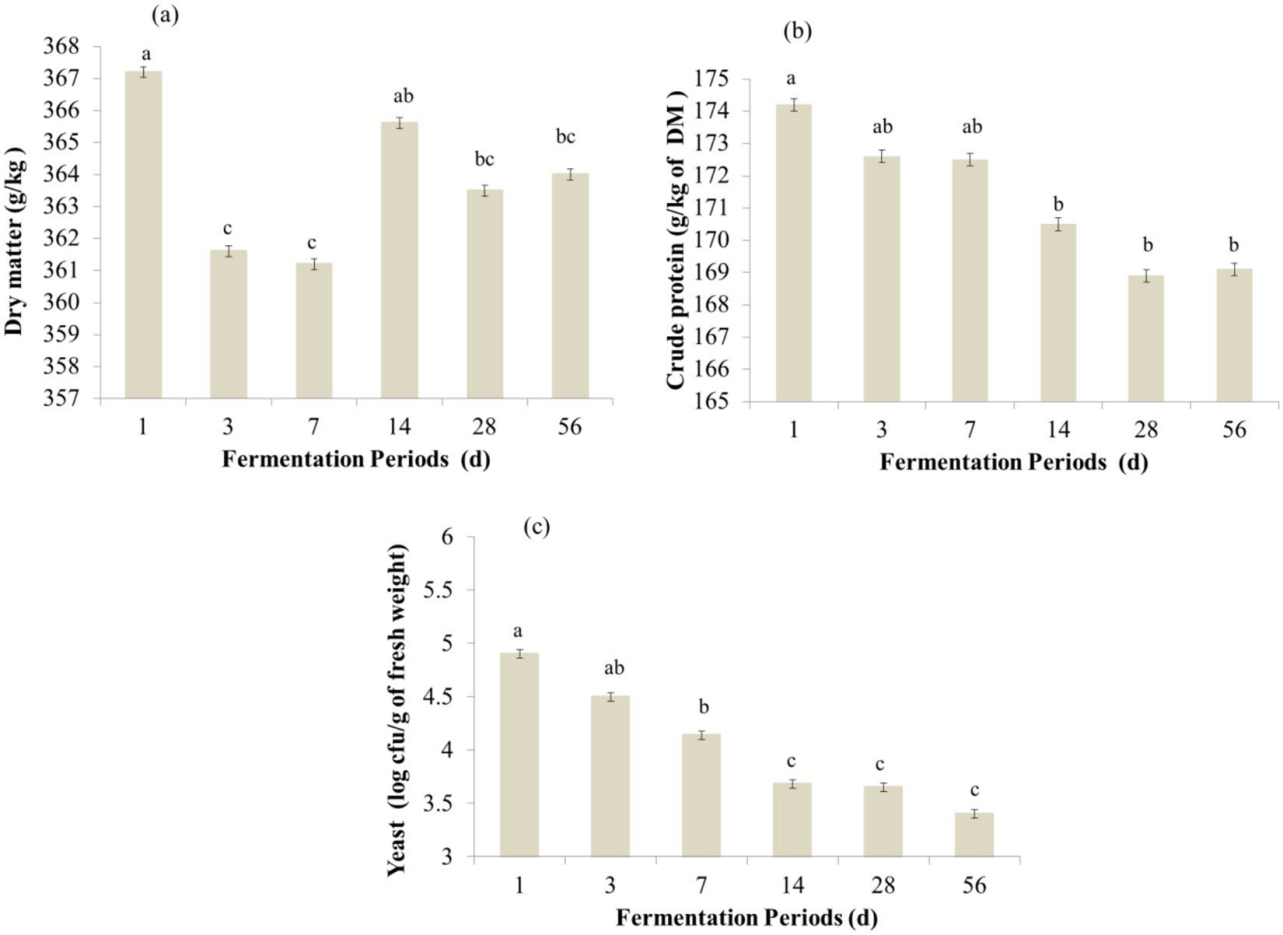
Effect of the fermentation period on the dry matter (**a**), crude protein (**b**) and yeast population (**c**) of alfalfa silages. ^a–c^Means followed by different letters are significantly different according to Tukey’s test (*P* < 0.05). CTRL = Control (without inoculant); CI = Commercial inoculant - Silobac; LPE = *Lactobacillus pentosus*; LP = *Lactobacillus plantarum*; PP = *Pediococcus pentosaceus*; LP + PP = *Lactobacillus plantarum* + *Pediococcus pentosaceus*.

The P-values and standard error of the mean of fermentation characteristics and microbial populations of alfalfa silages are shown in Table 2. There was an effect (*P* < 0.05) of I and P on DM content and yeast population. CP content was affected only by P. Lower DM were observed in CTRL and LP silages than the other silages (360.5 *vs*. 365.5 g/kg as fed). However, this difference was biologically insignificant (Fig. 1). LP, PP, and LP + PP silages had lower yeast counts than CTRL (3.76 *vs*. 4.46 log cfu/g of fresh weight) (Fig. 1). The CP content and yeast population were reduced during the fermentation period (Fig. 2).

**Table 2:** Significance (*P-values*) of the tested effects and the standard error of the mean (SEM) for the fermentation profile variables of alfalfa silages treated with inoculants at different fermentation periods.

Item	P-value			
	Inoculant	Period	Inoculant × Period	SEM
Dry matter	<0.001	<0.001	0.085	0.33
Crude protein	0.81	<0.001	0.06	0.38
pH	<0.001	<0.001	<0.001	0.06
WSC	<0.001	<0.001	<0.001	0.04
NH_3_-N	0.003	<0.001	<0.001	0.22
Lactic acid	<0.001	<0.001	<0.001	0.13
Acetic acid	<0.001	<0.001	<0.001	0.03
Propionic acid	<0.001	<0.001	<0.001	0.014
Butyric acid	<0.001	<0.001	<0.001	0.001
LAB^4^	<0.001	<0.001	<0.001	0.16
Enterobacteria	<0.001	<0.001	<0.001	0.21
Yeast	<0.001	<0.001	0.12	0.08
Mold	0.001	<0.001	<0.001	0.09

Item: WSC = Water-soluble carbohydrates; LAB = Lactic acid bacteria.

The I × P interaction affected (*P* < 0.05) the pH, WSC, NH_3_-N, LAB, enterobacteria and mold populations (Table 2). The characteristics of alfalfa silages as a function of microbial inoculant within each fermentation period are shown in Table 3.

**Table 3:** Average fermentation profile, chemical composition and microbial populations of alfalfa silages as a function of the microbial inoculant within each fermentation period.

Fermentation periods (days)						
Inoculant	1	3	7	14	28	56
**Lactic acid bacteria (log cfu/g of fresh weight)**						
CTRL	6.89^b^	8.42^b^	9.05^b^	9.20	9.42^a^	8.71^ab^
CI	7.47^ab^	9.79^a^	9.56^a^	9.32	9.32^a^	8.75^ab^
LPE	7.66^a^	9.94^a^	9.66^a^	9.38	9.20^a^	8.55^ab^
LP	7.81^a^	9.83^a^	9.52^a^	9.45	9.13^ab^	8.87^a^
PP	7.42^ab^	9.96^a^	9.38^a^	9.17	8.44^b^	7.94^b^
LP + PP	7.85^a^	9.91^a^	9.47^a^	9.65	9.19^a^	8.63^ab^
**Enterobacteria (log cfu/g of fresh weight)**						
CTRL	5.89	7.29^a^	6.84^a^	5.07^a^	3.98	4.48^a^
CI	5.66	6.04^ab^	5.04^b^	3.48^b^	3.13	2.79^ab^
LPE	6.03	6.52^ab^	4.80^b^	3.08^b^	3.30	2.45^b^
LP	6.10	5.48^b^	4.41^b^	2.85^b^	3.25	2.97^b^
PP	6.73	6.06^ab^	4.27^b^	2.56^b^	3.82	2.17^b^
LP + PP	6.79	6.71^ab^	5.00^b^	2.84^b^	3.15	2.52^b^
**Mold (log cfu/g of fresh weight)**						
CTRL	5.06	4.60^a^	4.23^a^	4.45^a^	3.84^a^	2.68
CI	5.11	3.83^b^	3.88^ab^	3.68^ab^	3.00^ab^	2.50
LPE	5.25	4.03^b^	3.59^ab^	3.45^ab^	2.49^b^	2.18
LP	5.05	3.69^b^	3.21^ab^	3.56^ab^	2.64^b^	2.43
PP	5.08	3.88^b^	2.97^b^	3.23^b^	2.43^b^	2.25
LP + PP	5.00	3.82^b^	2.99^b^	3.26^b^	2.32^b^	2.47
**Water-soluble carbohydrates (g/kg of DM)**						
CTRL	12.4^ab^	16.0^a^	9.8^a^	7.5^a^	3.0	3.4
CI	16.4^a^	3.7^b^	3.6^b^	4.1^b^	2.1	3.0
LPE	11.9^ab^	2.6^b^	5.0^b^	3.5^b^	2.6	3.6
LP	11.9^ab^	4.6^b^	3.0^b^	3.3^b^	2.6	3.6
PP	10.9^b^	4.1^b^	3.3^b^	4.1^b^	2.7	3.3
LP + PP	14.4^ab^	4.5^b^	3.3^b^	3.5^b^	2.9	3.2
**pH**						
CTRL	6.48	6.25^a^	5.34^a^	4.77^ab^	4.60^bc^	4.61^bc^
CI	6.46	4.81^bc^	4.58^c^	4.65^b^	4.74^ab^	4.72^b^
LPE	6.49	4.86^bc^	4.78^b^	4.79^ab^	4.80^a^	4.80^ab^
LP	6.46	4.77^c^	4.71^bc^	4.76^ab^	4.76^a^	4.88^a^
PP	6.52	4.88^bc^	4.62^c^	4.50^c^	4.48^c^	4.48^c^
LP + PP	6.51	4.95^b^	4.78^b^	4.81^a^	4.86^a^	4.82^ab^
**NH**_**3**_ **-N (g/kg of total nitrogen)**						
CTRL	20.2^ab^	30.4^ab^	46.0^ab^	54.7^a^	73.4^ab^	80.9^ab^
CI	10.6^b^	24.3^b^	59.9^a^	48.1^ab^	85.7^a^	78.0^ab^
LPE	26.3^a^	25.7^b^	58.9^a^	54.7^a^	81.8^ab^	85.0^a^
LP	14.0^ab^	30.3^ab^	40.0^b^	54.6^a^	71.2^b^	84.6^a^
PP	23.3^ab^	41.7^a^	46.3^ab^	51.6^ab^	59.9^b^	69.4^b^
LP + PP	27.6^a^	32.9^ab^	44.2^b^	40.4^b^	76.3^ab^	83.4^ab^

^a–c^Means within columns with different letters are significantly different according to Tukey’s test (*P* < 0.05).

Inoculant: CTRL = Control (without inoculant); CI = Commercial inoculant - Silobac; LPE = *Lactobacillus pentosus*; LP = *Lactobacillus plantarum;* PP = *Pediococcus pentosaceus*; LP + PP = *Lactobacillus plantarum* + *Pediococcus pentosaceus*.

The LAB population was not affected by I only at 14 d of fermentation. The lowest counts were observed in CTRL silages in the first week of fermentation. Viable LAB counts increased from 6.54 log cfu/g in fresh forage to greater than 9 log cfu/g of fresh weight in silages. The population subsequently declined slowly after this peak. However, the LAB peak value for the inoculated silages occurred as early as 3 d after fermentation, and the peak was observed only after 28 d in non-inoculated silages. Lower counts of LAB was observed in PP silages at 28 d than other silages, except LP. However, PP silages had lower counts than LP at 56 d.

The I × P interaction did not affect the enterobacteria population at days 1 and 28. CTRL silages had the highest population 7 and 14 d after fermentation. The new strains controlled more efficiently the population at the end of the fermentation resulting in lower counts of these microorganisms. The mold population was not affected by interaction at 1 and 56 d. CTRL silages had the highest population of this microorganism at day 3 and higher counts than PP and LP + PP at 7 and 14 d of fermentation.

Non-inoculated silages had higher concentrations of residual WSC from 3 to 14 d, but no differences between silages was observed after 14 d. The pH was not affected by I only on the first day of fermentation. CTRL silages had the highest pH 3 and 7 d after ensiling. Inoculated silages had the highest pH decline rates in the first week of fermentation. PP resulted in the lowest pH values from 14 d of fermentation.

Inoculants affected the concentration of NH_3_-N at all fermentation periods. Lower values were observed for LP and PP silages at 28 d compared to CI (65.5 *vs*. 85.7 g/kg TN). PP silages had lower concentrations than LP and LPE (69.4 *vs*. 84.8 g/kg TN) after 56 d of fermentation.

The I × P interaction also affected the production of lactic, acetic, propionic and butyric acids (*P* < 0.05) (Table 2). The acid concentrations as a function of microbial inoculant within each fermentation period are shown in Table 4. An I × P effect was observed on the concentrations of lactic acid at 3, 7 and 28 d. CTRL silages had the lowest concentrations at 3 and 7 d. PP silages had the highest lactic acid values on day 28 of fermentation (40.6 *vs*. 34.9 g/kg DM).

**Table 4:** Average organic acid production of alfalfa silages as a function of the microbial inoculant within each fermentation period.

Fermentation periods (days)						
Inoculant	1	3	7	14	28	56
**Lactic acid (g/kg of DM)**						
CTRL	7.7	6.3^b^	18.6^b^	26.5	34.4^b^	33.2
CI	7.6	32.0^a^	36.9^a^	29.6	30.0^b^	34.2
LPE	9.6	30.5^a^	30.5^a^	27.2	34.4^b^	37.9
LP	7.7	27.8^a^	35.3^a^	34.3	32.9^b^	39.9
PP	9.3	27.9^a^	32.9^a^	33.5	40.6^a^	37.3
LP + PP	7.8	25.3^a^	34.4^a^	29.3	38.5^b^	42.0
**Acetic acid (g/kg of DM)**						
CTRL	3.7	5.0^ab^	8.7^a^	6.4^b^	11.3^a^	8.9^ab^
CI	5.2	4.1^ab^	7.0^ab^	6.8^b^	8.3^ab^	8.2^ab^
LPE	5.9	5.3^ab^	7.2^ab^	6.1^b^	9.1^ab^	10.4^a^
LP	4.3	4.7^ab^	6.3^ab^	9.3^a^	7.3^b^	11.1^a^
PP	5.5	4.0^b^	5.1^b^	6.4^b^	6.5^b^	6.4^b^
LP + PP	4.5	6.8^a^	6.5^ab^	8.2^b^	9.2^ab^	10.7^a^
**Propionic acid (g/kg of DM)**						
CTRL	2.73	6.33^a^	2.66^a^	0.92	0.87	1.08
CI	2.25	5.76^a^	0.98^b^	1.06	0.96	1.05
LPE	2.52	3.08^b^	2.79^a^	0.96	0.94	1.06
LP	2.96	1.79^c^	1.09^b^	0.72	0.97	1.06
PP	2.69	0.93^c^	1.12^b^	0.98	1.04	1.14
LP + PP	2.62	1.02^c^	0.87^b^	0.90	0.90	1.13
**Butyric acid (g/kg of DM)**						
CTRL	0.22	0.30	0.58^a^	0.32	0.29	0.32
CI	0.23	0.31	0.48^b^	0.30	0.31	0.23
LPE	0.24	0.42	0.33^b^	0.25	0.31	0.45
LP	0.24	0.36	0.40^b^	0.19	0.45	0.31
PP	0.22	0.40	0.27^b^	0.23	0.45	0.43
LP + PP	0.26	0.31	0.33^b^	0.27	0.30	0.46

^a–c^Means within columns with different letters are significantly different according to Tukey’s test. (*P* < 0.05).

Inoculant: CTRL = Control (without inoculant); CI = Commercial inoculant - Silobac; LPE = *Lactobacillus pentosus*; LP = *Lactobacillus plantarum;* PP = *Pediococcus pentosaceus*; LP + PP = *Lactobacillus plantarum* + *Pediococcus pentosaceus*.

The interaction did not affect the acetic acid concentration only at 1 d after fermentation. LP + PP and CTRL silages had higher concentrations than PP silages at days 3 and 7, respectively. The highest concentrations were observed in LP silages (9.3 g/kg DM) at day 14. Lower values were observed in LP and PP silages compared to CTRL (6.9 *vs*. 11.3 g/kg DM) at 28 d. PP silages had the lowest acetic acid production comparing to the new strains at 56 d.

Propionic acid was affected by the interaction at 3 and 7 d after fermentation. CTRL and CI silages had the highest concentration at day 3, and CTRL and LPE had the highest concentrations at day 7. There was a slight increase in butyric acid concentration in CTRL silages only 7 days after fermentation.

The chemical composition and IVDMD of alfalfa silages at 56 d of fermentation are presented in Table 5. The DM, CP, NDF and ADF of silages at 56 d of fermentation were unaffected (*P* > 0.05) by I and averaged 364 g/kg as fed, 169.2, 388.0 and 259.0 g/kg DM, respectively. Silages inoculated with PP had higher coefficients of digestibility than LP silages (0.644 *vs*. 0.611).

**Table 5:** Average (with the respective standard error of the mean and ANOVA-based *P-value*) chemical composition and coefficient of the *in vitro* dry matter digestibility of alfalfa silages treated with microbial inoculants at 56 d of fermentation (g/kg DM, unless otherwise stated).

Item	Alfalfa silage day 56						SEM	*P-value*
	CTRL	CI	LPE	LP	PP	LP + PP		
Dry matter (g/kg)	358.0	369.0	366.9	362.9	364.4	363.0	0.11	0.09
Crude protein	171.7	171.5	168.8	167.9	167.2	168.1	1.33	0.31
NDF^3^	383.0	346.1	324.1	353.7	369.0	371.7	0.61	0.06
ADF^4^	265.1	255.8	258.2	253.6	256.5	268.0	0.28	0.72
IVDMD^5^	0.634^ab^	0.614^ab^	0.625^ab^	0.611^b^	0.644^a^	0.613^ab^	0.36	0.02

^a,b^Means within rows with different letters are significantly different according to Tukey’s test (*P* < 0.05).

Item: NDF = Neutral detergent fiber; ADF = Acid detergent fiber; IVDMD = Coefficient of *in vitro* dry matter digestibility.

CTRL = Control (without inoculant); CI = Commercial inoculant - Silobac; LPE = *Lactobacillus pentosus*; LP = *Lactobacillus plantarum;* PP = *Pediococcus pentosaceus*; LP + PP = *Lactobacillus plantarum* + *Pediococcus pentosaceus*.

## Discussion

Silage is a very complex fermentation matrix that exhibits variability in natural microbiota, chemical composition and nutrients, such as WSC and the nitrogenous components available for microbes^20^. The occurrence of desirable silage fermentations is guided by the amount and type of microorganisms present in the plant and the DM content, buffering capacity and WSC of the forage^3^.

The average WSC of raw alfalfa was lower than the 40–60 g/kg DM recommended by Mahanna^21^ as adequate for the occurrence of good fermentation of silage. However, studies on alfalfa silage also reported WSC between 10 and 40 g/kg DM^22,23^. The LAB counts were higher than the minimum established by Muck^24^ (5.0 log cfu/g fresh weight) as adequate for the occurrence of good fermentation of silage.

The faster increase in LAB counts observed in inoculated silages in early fermentation indicated that the LAB strains were competitive among the epiphytic communities. Microbial changes during this phase in successfully fermented silages are primarily the result of the disappearance of enterobacteria and the development of a dominant LAB population. The speed of this shift closely correlates with the rate of pH decline and lactic acid production^25^. The reduction in the LAB population after the peak in all assessed silages was expected because low pH and the lack of fermentable substrates result in bacterial death^26^.

The reduction of pH is related to the conservation of the ensiled material. The fast initial acidification promotes a decrease in the enzyme-mediated proteolytic activity of the plant itself and controls the growth of enterobacteria and clostridia, which grow until an inhibitory concentration of non-dissociated acids and/or sufficiently low pH are reached^27^.

In our study, the acidification induced by epiphytic bacteria fermentation in the CTRL silages reached the similar values of inoculated silages pH after 14 d. The highest pH values on days 3 and 7 in the CTRL silages reflected the low epiphytic LAB counts and its low efficiency in initiating fermentation and controlling undesirable microorganisms compared to the LAB strains, as suggested by Davies *et al*.^28^. The final pH values of all the silages were within the range of 4.48–4.88, which is considered adequate for legume silages, which usually stabilize when the pH drops to between 4.5 and 4.9^29^.

The changes in the WSC contents are related to the use of these carbohydrates by bacteria as substrates for growth, which results in the synthesis of primarily lactic acid^30^. As expected, the WSC concentrations of all silages were reduced during the fermentation. The highest residual WSC content in the CTRL silages in the first week of fermentation reflects the lower fermentation intensity in these silages, as evidenced by the lower LAB counts and lactic acid concentrations and higher pH values.

Zielińska *et al*.^6^ found that some LAB strains developed more intensively in ensiled plants because of their role in the partial hydrolysis of starch, cellulose and xylans. This capacity may explain the lowest pH values of the PP silages with similar residual WSC concentrations at day 28 of fermentation, which was reflected by the highest conversion of substrate into lactic acid in these silages during the same period.

The reduction of CP content during the fermentation process was due to the plant and microbial proteolytic processes in the ensiled material, which change the nitrogenous compounds in silages and results in an increase in soluble N and NH_3_-N^31^, as observed in our study. According to Langston *et al*.^32^ proteolysis results in the formation of peptides and amino acids. The NH_3_-N formation is a reflection of amino acid deamination, which characterizes the end of a putrefactive process. The NH_3_-N concentrations were different between silages in our study, but no differences in CP concentrations were observed between inoculants, which suggests that the production of NH_3_-N resulted from different intensities of the deamination of free amino acids in the material.

The higher NH_3_-N concentrations of silages inoculated with LPE and LP strains compared to PP on the last day of fermentation were reflected by the higher pH and acetic acid concentrations in these silages, which indicated the growth of undesirable microorganism. Kung^10^ and Oliveira *et al*.^1^ reported that *P*. *pentosaceus* strain inoculation did not affect NH_3_-N concentrations in silage, which may be related to its slower growth rate than other bacteria, but this effect was not observed in our study.

The main acids identified in the silages are acetic, butyric and lactic because these acids represent the highest concentrations of acids^33^. Kung *et al*.^31^ demonstrated that lactic acid was generally found at the highest concentration in silages during the ensiling process and contributed the most to the decrease in pH during fermentation because it is approximately 10 to 12 times stronger than the other major acids. The concentrations of lactic acid in the silages were 20 to 40 g/kg DM, which are the concentrations commonly found in legume silages that were also reported by these authors.

The lower production of lactic acid in CTRL silages in the first week of fermentation reflects the lower LAB counts and their ability to dominate the fermentation, as discussed previously. Muck and Kung^5^ found that silages treated with homofermentative bacteria resulted in lower silage pH compared to untreated silages because of the greater production of lactic acid, which may be more evident in legume than corn silage.

Although lower LAB counts were observed in the PP silages at day 28, higher lactic acid values were produced, which shows the efficiency of substrate utilization and the persistence of acidification of the strain. PP silages had lower pH than other inoculated silages at day 56 of fermentation with the same lactic acid concentration, which may be attributed to the lower production of NH_3_-N and acetate in these silages and the reduced buffering effects of these compounds on the ensiled material^34^.

The contents of acetic and butyric acids are primary negative indicators of the quality of the fermentation process and also correspond to silages that showed marked losses of dry matter and energy during fermentation. Lower concentrations of acetic acid in PP silages at the end of the fermentation period may result in higher DM recovery, and it indicates the predominance of homolactic fermentation compared to the LP, LPE, and LP + PP silages. The highest concentrations of propionic acid at 3 and 7 d in CTRL silages may have resulted from secondary fermentations, especially because the concentrations of lactic acid were lower in this silage during this period.

Enterobacteria are generally the second most numerous bacterial group of the epiphytic microbiota active in the silo. Their population and rate of decline are used as indicators of silage quality because these microorganisms are main competitors with LAB for available sugars and result in gas losses and a reduction in the nutritional value of the silages^30^.

The dominance of LAB, the faster drop in the pH induced by higher lactic acid production and the synergistic effects of the acids produced during the fermentation in our study resulted in the reduction of enterobacteria counts in all inoculated silages until 14 days of fermentation. The same reasons are attributed to the reducing of yeast population throughout the fermentation periods.

The studied variables did not affect the chemical composition of silages at 56 d of fermentation, which suggests that the attendant improvements in silage characteristics are often lacking even when the concentrations of supposedly explanatory metabolites increase in response to bacterial inoculation. This effect may occur because the explanatory metabolites only explain a fraction of the variability in the response to an inoculant^35^. The measurement of IVDMD is used to analyze the nutrient digestibility of feed ingredients^15,36^.The values of IVDMD in our study were similar to Nadeau *et al*.^37^ and Rodrigues *et al*.^23^, also in alfalfa silage.

In conclusion, all of the novel strains tested had a positive effect on at least one chemical property of the silage during the fermentation process. However, the addition of *P*. *pentosaceus* alone had a positive influence on all of the evaluated parameters and changed the characteristics of the silages; particularly, the strain enhanced the lactic acid content and decreased the pH, deteriorating microorganisms, and NH_3_-N and acetic acid concentrations, which resulted in a better silage quality that surpassed the commercial inoculant. We suggest that this strain has potential for use as a silage inoculant, but it must be tested in different forages and in combination with other additives, such as heterofermentative bacteria or chemical additives. The results obtained at the laboratory scale must also be confirmed under more practical conditions.

## Supplementary information

Characterization of lactic acid bacteria isolated from non-wilted and wilted alfalfa silages
