# Supplementary material for: Novel lactic acid bacteria strains as inoculants on alfalfa silage fermentation
Source: Sci Rep. 2019 May 29;9:8007. doi: 10.1038/s41598-019-44520-9 (PMC6541639; doi:10.1038/s41598-019-44520-9)
Supplement: Supplementary file 1 — Characterization of lactic acid bacteria isolated from non-wilted and wilted alfalfa silages [file 41598_2019_44520_MOESM1_ESM.pdf]

Novel lactic acid bacteria strains as inoculants on alfalfa silage fermentation

Mariele Cristina Nascimento Agarussi[1*], Odilon Gomes Pereira[1*], Rosinea Aparecida de Paula[1], Vanessa Paula da Silva[1], João Paulo Santos Roseira[1], Fabyano Fonseca e Silva[1]

[1]Department of Animal Science, Federal University of Vicosa, Viçosa, 36570-000 Brazil.

*Correspondence and requests for materials should be addressed to O.P. (odilon@ufv.br)

Table S1. Characterization of lactic acid bacteria isolated from non-wilted and wilted alfalfa silages

Isolate ID		3.7 E	14.7 SE	14.15 SE
Identification		*Lactobacillus plantarum*	*Lactobacillus pentosus*	*Pediococcus pentosaceus*
Access number		MH924275	MH924298	MH924301
Control condition	MRS/37°C	+++	++++	+++
Temperature (°C)§	15	+	+	+
	45	-	+	+
pH§	3.5	+	+	+
	4	+	+	+
	4.5	+	+	+
	8.5	+	+	+
g/L NaCl§	40	+	+	+
	65	+	+	+
CO_2*		Ho	Ho	Ho
1†	Radius of inhibition‡	++	++	++
2†		++	++	++
3†		++	++	++
pH	After 24h	4.18	4.12	4.25
Metabolites (g/kg of DM)	Lactic acid	14.9	15.1	13.3
	Acetic acid	3.1	2.8	1.9
	Propionic acid	0	0.1	0.2

§: Growth measured by optical density (630 nm) - = Absence of growth; + = Presence of growth; *Ho: Homofermentative; He: Heterofermentative; †: Indicator microorganisms of the antimicrobial test. 1- *Listeria monocytogenes* 19117; 2- *Listeria monocytogenes* 7644; 3- *Escherichia Coli*; ‡: (mm) : - (absence of inhibition halo), + (> 4 and ≤ 15), ++ (> 15 and ≤ 30), +++ (> 30).
